# The Sum Score Model: Specifying and Testing Equally Weighted Composites Using Structural Equation Modeling

**DOI:** 10.1017/psy.2024.5

**Published:** 2025-01-03

**Authors:** Florian Schuberth, Tamara Schamberger, Ildikó Kemény, Jörg Henseler

**Affiliations:** 1 Department of Design, Production & Management, University of Twente, Enschede, The Netherlands; 2 Faculty of Business Administration and Economics, University of Bielefeld, Bielefeld, Germany; 3 Department of Digital Marketing, Corvinus University of Budapest, Budapest, Hungary; 4 Nova Information Management School, Universidade Nova de Lisboa, Lisbon, Portugal

**Keywords:** composite model, equal weights, full transmission, Henseler–Ogasawara specification, sum scores

## Abstract

In principle, structural equation modeling (SEM) is capable of emulating all approaches based on the general linear model. Yet, modeling sum scores in a structural equation model is not straightforward. Existing approaches to studying sum scores in a structural equation model are limited in terms either of model specification or of model assessment. This paper introduces a specification to SEM that allows for directly modeling sum scores and that overcomes existing approaches’ limitations in dealing with sum scores in the SEM context. The sum score model we present builds on the recently proposed refined Henseler–Ogasawara (H–O) specification of composites. It allows us to estimate models with sum scores in an integrative way. It can mimic the results of existing approaches and provides a means of assessing whether a sum score fully transmits the effects of or on the variables that make up the sum score. In addition, it allows for taking into account random measurement error in the variables that form the sum score. Consequently, this model specification offers researchers an improved way of judging and defending the use of sum scores empirically and conceptually.

## 1 Introduction

Sum scores have a long tradition in the social sciences (Kuder & Richardson, 1937; Richardson, 1936; Traub, 2005). They are calculated as the sum of observed variables and constitute a straightforward way of creating scores of theoretical constructs (Nunnally & Bernstein, 1994). Due to the ease of calculating them, sum scores are very popular in various disciplines, including psychology (McNeish & Wolf, 2020), biology (Cheniti et al., 2017), medical research (Wang & Reeve, 2021), and physics (Hardt et al., 2018). Arguably, sum scores owe their popularity to the fact that using them to build construct scores ensures comparability and simplifies the reproducibility of different studies (Widaman & Revelle, 2023). Further, the popularity of sum scoring is reflected in the ample use of various metrics that build on sum scores, such as Cronbach’s 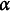 (Cronbach, 1951; Sijtsma, 2009), McDonald’s (1999) 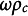 , and the heterotrait-monotrait ratio of correlations (HTMT/HTMT2, Henseler et al., 2015; Roemer et al., 2021; Rönkkö & Cho, 2022).

Sum scores are used for various reasons. For instance, they are used in procedures such as item parceling (Little et al., 2002). Moreover, sum scores are often used as “fallible (i.e., imperfect) estimates of the relative position of individuals on the dimension implied by the sum scores and pretend to be nothing more” (Widaman & Revelle, 2023, p. 794). However, a sum score also constitutes a special type of composite, i.e., a weighted linear combination of variables, and thus can be used to represent theoretical constructs (Cohen et al., 1990; Grace & Bollen, 2008; Saris & Gallhofer, 2014). “If a construct is defined in a way such that the building of a sum score maps on this definition well (Lundberg et al., 2021), then its use is appropriate” (Edelsbrunner, 2022, p. 3). For example, job satisfaction can be considered as the sum of different aspects, which include salary and working hours, opportunities for advancement, job security, autonomy in doing the work, social contacts, and usefulness of the job for society (Saris & Gallhofer, 2014, Chapter 1). In this case, a sum score would summarize the effects of or on the variables forming the sum score, i.e., the collective effects of or on the sum score’s variables. In the literature, such a summary effect, i.e., the effect of or on the composite, is also known as the sheaf coefficient (Heise, 1972).

A widely used approach in the social and behavioral sciences for studying relationships between variables is structural equation modeling (SEM, Bollen, 1989). It provides researchers with several means by which to assess models, such as the 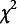 -test (Jöreskog, 1969), various fit indices (Hu & Bentler, 1998), and information criteria (Bozdogan, 1987). Moreover, SEM is a very versatile and holistic framework, which can in principle emulate all approaches based on the general linear model (Graham, 2008). Hence, it should be able to seamlessly incorporate sum scores in SEM. In fact, several suggestions have been made. Specifically, analysts can use a two-step approach to include sum scores in a structural equation model or can rely on approaches to model sum scores in SEM, such as the one-step approach (e.g., Grace & Bollen, 2008) and the pseudo-indicator approach (Rose et al., 2019).

Unfortunately, the existing approaches that enable incorporating sum scores in SEM all come with limitations. The two-step approach does not take the formation of the sum score into account and thus omits the sum score’s components from the actual model. This prevents researchers from exploiting the full capabilities of SEM, such as applying the direct maximum likelihood approach for dealing with missing values in the variables making up the sum score (e.g., Allison, 2003). Similarly, the one-step approach (Grace & Bollen, 2008) can only model a sum score as a predictor of other variables in the model (MacCallum & Browne, 1993). Thus, this approach is limited in its flexibility for modeling sum scores. Further, although the pseudo-indicator approach permits the flexible modeling of sum scores in SEM, there is currently no available guidance on how to model a sum score that fully transmits the collective effects on its components. As a result, the current approaches do not allow for flexible modeling of sum scores in SEM or miss opportunities to assess them.

To overcome the limitations of the existing approaches to dealing with sum scores in a structural equation model, we introduce a new way of specifying sum scores in SEM: the sum score model. This model is a special case of the recently introduced Henseler–Ogasawara (H–O) specification of composites (Henseler, 2021;Schuberth, 2023; Yu et al., 2023). It allows researchers to flexibly integrate sum scores into a larger model that also includes other variables. Moreover, it is possible to assess whether a sum score fully transmits all collective effects of or on the variables that make up the sum score. This can be useful when a sum score is used to model a theoretical construct (Grace & Bollen, 2008). Further, we present two ways of accounting for random measurement error in the sum score model to avoid attenuation bias in the parameter estimates. Finally, as our proposed sum score model is based on the refined H–O specification, it is straightforward to replace a sum score with a composite whose weights are freely estimated.

The remainder of this article is structured as follows. Section 2 presents the existing approaches to specifying sum scores in SEM. Particularly, we discuss the various approaches’ advantages and disadvantages. In Section 3, we introduce the sum score model based on the refined H–O specification as an alternative, more flexible approach to model sum scores in SEM. Additionally, we show how to relax the assumption of the original H–O specification in the sum score model according to which the sum score fully transmits all collective effects of or on its components. Further, we present two ways of accounting for random measurement errors in the sum score model. By means of three illustrative examples, Section 4 compares the results of our sum score model with those of existing approaches. This section also highlights the sum score model’s capabilities. Finally, in Section 5, we close the paper with a discussion.

## 2 Existing approaches to dealing with sum scores in SEM

Several approaches have been proposed for dealing with sum scores in SEM. They can be divided into approaches that *include* sum scores and approaches that *model* sum scores in a structural equation model.

### 2.1 Approach to include sum scores

The *two-step approach* can be considered the classical approach for dealing with sum scores in SEM. As its name suggests, it includes a sum score in the model following two steps. In the first step, the sum score is calculated before the actual analysis, i.e., the variables forming the sum score are simply summed up. Subsequently, in the second step, the sum score is used as a new observed variable to replace the original variables in a structural equation model.^1^

On the one hand, the two-step approach is very easy to implement. On the other hand, the fact that the creation of the sum score is not modeled and, thus, the variables making up the sum score are not included in the final model, holds major disadvantages. Particularly, it is not possible to assess whether the sum score properly summarizes the collective effects of or on its components. In other words, it is not possible to examine whether the sum score fully transmits the effects of or on its components. Hence, this approach misses opportunities for model assessment. Further, researchers studying sum scores might not benefit from all of SEM’s capabilities. For instance, researchers cannot use the direct maximum-likelihood approach (e.g., Allison, 1987, 2003) also known as full information maximum likelihood approach to deal with missing values in the variables making up a sum score and need to rely on alternatives such as the two-stage maximum likelihood approach (e.g., Chen et al., 2020; Savalei & Bentler, 2009).

### 2.2 Approaches to model sum scores

Researchers have started to develop approaches that can be used to model sum scores in SEM. In contrast to the two-step approach, these approaches model sum scores in a single step, thus considering the creation of a sum score within the model. Arguably, the most straightforward approach to model sum scores is the *one-step approach*. This approach models the sum score as a latent variable in a causal-formative measurement model (e.g., Bollen & Lennox, 1991). The effects of all indicators on the latent variable are fixed to one and the variance of the latent variable’s error term is fixed to zero. Consequently, the latent variable becomes an observed variable, i.e., the sum score. For more information on the one-step approach, we refer the interested reader to Grace and Bollen (2008).

The one-step approach allows for directly modeling sum scores in a structural equation model, thus overcoming the two-step approach’s limitations. It takes the creation of the sum score in the model into account, and thus allows researchers to employ the direct maximum likelihood approach to deal with missing values in the observed variables that make up the sum scores. Further, it can be used to assess whether a sum score fully transmits the effects of the variables that make up the sum score on some outcome variables (Grace & Bollen, 2008). However, this approach also has its limitations. For instance, it does not allow for modeling effects of other variables on the sum score, i.e., modeling the sum score as an outcome variable in the structural model is not possible. In such cases, the model would not be identified (MacCallum & Browne, 1993). Hence, it is not possible to model and assess whether a sum score properly summarizes the collective effects of other variables on the variables making up the sum score, i.e., whether the sum score fully transmits the effects on the variables that make up the sum score. Similarly, since this approach always models the sum score as a dependent variable, it is not possible to specify covariances between the sum score and other exogenous variables of the model. A putative solution would be to model covariances between the error term of the latent variable and other exogenous variables. However, since the error term’s variance is fixed to zero, this would be a fruitless endeavor.

A more flexible approach to model sum scores is the *pseudo-indicator approach* (Rose et al., 2019). This approach takes advantage of the fact that a sum score is the sum of observed variables. Specifically, one of the observed variables becomes the pseudo-indicator, which is expressed as the difference between the sum score and the remaining observed variables. Thereby, the sum score is modeled as a latent variable with a single indicator, i.e., the pseudo-indicator. To ensure that the sum score is in fact the sum of its variables, the variance of the resulting error term of the pseudo-indicator needs to be fixed to zero. In addition, the effects of the remaining observed variables on the pseudo-indicator are fixed to minus one. Consequently, the latent variable becomes the sum score. Finally, the covariances between the remaining observed variables and the sum score (and usually also the covariances between the remaining observed variables and potential other exogenous variables of the model) are specified. For more details on the pseudo-indicator approach, we refer the interested reader to Rose et al. (2019).

The pseudo-indicator approach is an elegant way of flexibly modeling sum scores in SEM. It overcomes the drawbacks of the one-step approach, i.e., it can model a sum score as a predictor or an outcome variable, and it allows for specifying covariances between sum scores and other exogenous variables of the structural equation model. However, the extant literature on the pseudo-indicator approach currently lacks guidance on how to model a sum score that fully transmits the collective effects on its components. Originally, the pseudo-indicator approach models a sum score in such a way that the resulting model is equivalent to the target model. The target model is the model that includes the sum score and other variables of interest but not the observed variables that form the sum score. For this purpose, the constraints created by including the sum score’s observed variables are removed. Thereby, it is ensured that the model-implied variance-covariance matrix of the target model remains unaffected by the inclusion of the sum score’s observed variables. Specifically, various covariances between the sum score’s observed variables, i.e., all observed variables that make up the sum score, except the pseudo-indicator, and other exogenous variables of the model are specified. Although Rose et al. (2019) mention the possibility of fixing these covariances to zero, which allows one to assess whether a sum score fully transmits the collective of its components, they do not currently provide any information on how to fix these covariances to ensure that the sum score fully transmits the collective effects on its components. We must, therefore, conclude that this approach is currently limited in its ability to assess whether a sum score properly summarizes the collective effects.

## 3 A new approach for modeling sum scores in SEM

In this section, we present a new approach for modeling sum scores in SEM: the sum score model. The sum score model is based on the refined Henseler–Ogasawara (H–O) specification (Schuberth, 2023; Yu et al., 2023). As sketched by Henseler (2021), the H–O specification to flexibly model composites in SEM uses the idea, which Ogasawara (2007) also introduced in the context of canonical correlation analysis, of expressing the relationship between a composite and the variables making up the composite in terms of (composite) loadings. This explains the name of this specification. Subsequently, the H–O specification has been elaborated (Schuberth, 2023) and refined to reduce its complexity (Yu et al., 2023). Since a composite is a weighted linear combination of other variables (e.g., Cohen et al., 1990; Edwards & Bagozzi, 2000), a sum score can be viewed as a special type of composite, namely a composite created with unit weights. Therefore, the H–O specification seems to be well suited for modeling sum scores in SEM. In the following subsections, we present the refined H–O specification and modifications of it that are potentially relevant to researchers dealing with sum scores. Specifically, in the next subsection, we present the refined H–O specification for composites, where the composite weights are free model parameters. Next, we show the parameter constraints necessary to obtain unit weights and thus sum scores, i.e., the sum score model. Further, we demonstrate in the sum score model how to relax the assumption of the original refined H–O specification that all effects on or of the variables that make up the composite are fully transmitted by the composite. In this way, the sum score model can achieve the same results as the pseudo-indicator approach. Finally, in the last subsection, we show how random measurement error in the variables that make up a sum score can be taken into account in the sum score model. To present the H–O specification and the sum score model, we use the SEM framework proposed by Jöreskog (1970); see also Jöreskog (1978). In particular, we assume that the observed variables follow a multivariate normal distribution, and for the sake of simplicity, we assume that the observed variables are mean centered.

### 3.1 Modeling composites using the refined H–O specification

The starting point is a set of observed variables 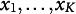 with variance-covariance matrix 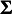 that composes a composite 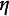 : 
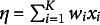
 .^2^ The H–O specification exploits the fact that *K* distinct composites can be extracted from these observed variables. For this reason, in addition to the composite of interest 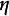 , 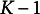 further composites are extracted from the set of observed variables, as Equation (1) shows. 
(1)

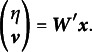

The additional composites 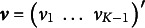 are referred to as *excrescent variables* and together with the composite of interest 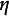 they span the entire space of the observed variables. The square matrix 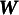 of dimension *K*, in its columns, contains the weights to form the composite of interest and the 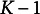 excrescent variables.

As is known from principal component analysis, the relations between composites and their observed variables can be expressed both by means of weights and by means of composite loadings: 
(2)

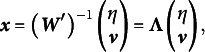

where the square matrix 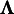 of dimension *K* contains the loadings of the composite 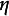 and the excrescent variables 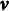 in its columns. As Equation (2) shows, the weights can, in principle, be obtained as the elements of the inverse of the transposed composite loading matrix: 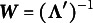 .

To ensure that the H–O specification is identified and that the loading matrix is invertible, some parameters need to be fixed (Schuberth, 2023; Yu et al., 2023). To determine the scale of the composites, i.e., the composite of interest and the excrescent variables, one loading can be fixed for each composite. Usually, these composite loadings are set to one. Each observed variable may only be used once for scaling purposes. In addition, we need to determine how the excrescent variables are extracted from the observed variables. In general, there are various ways to do this, as long as the identification of the parameters is ensured. In this study, we use the parameterization of the refined H–O specification (Yu et al., 2023). Therefore, only two observed variables are allowed to load on each excrescent variable, and we have to ensure that no excrescent variables are connected to exactly the same observed variables. Further, no observed variable is allowed to be related to more than two excrescent variables. Finally, the excrescent variables are allowed to correlate freely with each other, but they have to be uncorrelated with the composite of interest 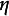 . This ensures that the variance-covariance matrix of the observed variables that form a composite can be perfectly reproduced, i.e., no constraints are imposed on the variance-covariance matrix 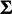 of the composite’s observed variables. Furthermore, as the weights are freely estimated in this H–O specification, the composite of interest 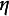 must not be isolated, i.e., it must have a relationship, e.g., a path coefficient or covariance, with at least one other variable of the model besides its observed variables (see also Dijkstra, 2017). This is supported by the fact that the H–O specification based on free weights without additional variables related to the composite of interest shows a negative number of degrees of freedom if more than one variable forms the composite. Similarly, this highlights that the freely estimated weights are context specific, i.e., the weights depend not only on the variables that make up the composite but also on the other variables of the model, including their metrics (Heise, 1972). The same holds for the one-step approach if the weights are freely estimated.

**Figure 1:**
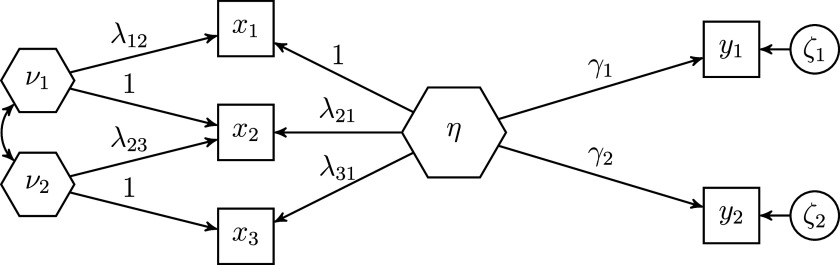
Example of the refined Henseler–Ogasawara specification with free weights.

Figure 1 shows an example of the refined H–O specification for a composite 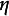 made up of three observed variables 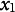 , 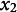 , and 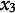 . For the sake of clarity, the variances of the exogenous variables are not shown in this figure. In this example model, the six collective effects of the observed variables 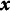 making up the composite of interest 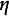 on the two outcome variables 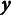 are calculated as: 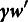 , where the column vector 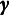 contains the two summary effects of the composite on the outcome variables, and the column vector 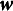 contains the three weights used to form the composite of interest. We provide the derivation of the collective effects in Appendix A.1.

### 3.2 The sum score model based on the refined H–O specification

**Figure 2:**
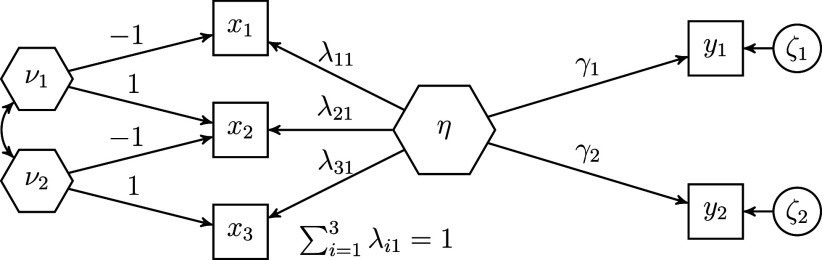
Example of a sum score model based on the refined Henseler–Ogasawara specification.

A sum score is a special type of composite, namely a composite created using unit weights. To account for this fact in the refined H–O specification, further constraints have to be imposed to ensure that the weights are equal to one. Therefore, we first change the scaling condition for the composite of interest. Instead of fixing one loading of the observed variables to one, we fix the sum of the loadings to one. In the context of latent variable models, this approach is also known as effects coding (e.g., Klopp & Klößner, 2021; Little et al., 2006). Next, we fix the loadings of each excrescent variable in such a way that their sum equals zero, e.g., by fixing one loading to one and the other loading to minus one. Figure 2 depicts an H–O specification in which the composite of interest 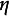 constitutes a sum score, i.e., it depicts a sum score model. For the sake of clarity, the variances of the exogenous variables are not depicted in this figure. It is noted that, in contrast to the refined H–O specification with free weights, it is no longer necessary that the sum score be connected to other variables of the model as the weights are fixed in the sum score model.

To demonstrate that these constraints lead to a sum score, we recall that in the H–O specification the weights are obtained as the inverse of the transposed composite loading matrix. Thus, the product of the transposed composite loading matrix and the weight matrix equals the identity matrix of dimension 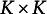 : 
(3)

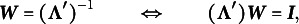

where the first column of 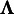 contains the composite loadings of 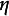 and the remaining columns contain the composite loadings of 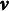 . Similarly, the first column of 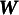 contains the weights to form 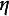 , while the remaining columns contain the weights to form 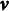 . Consequently, the product of the composite of interest’s loadings and its weights must equal one: 
(4)

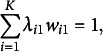

Similarly, the product of each individual excrescent variable’s loadings and the weights of the composite of interest must be equal to zero: 
(5)

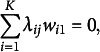

where *j* refers to the excrescent variables, i.e., 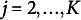 . In addition, each excrescent variable is related to exactly two observed variables 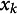 and 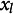 . Therefore, Equation (5) simplifies to: 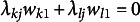 . As the two loadings are fixed in such a way that they sum up to zero, the two weights 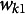 and 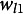 must be equal. Moreover, no observed variable is related to more than two excrescent variables, and no excrescent variables are connected to the same observed variables. This implies that the weights forming the composite of interest are all equal, i.e., 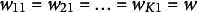 . Consequently, Equation (4) becomes: 
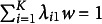
 . Since the sum of the composite of interest’s loadings is constrained to be equal to one for identification purposes, the weights that form the composite of interest are all equal to one: 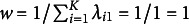 .^3^ Consequently, the composite of interest constitutes a sum score.

The sum score model we have presented has several advantages. For instance, it provides a versatile way of integrating sum scores into structural equation models. Similar to the pseudo-indicator approach, it offers us the flexibility to model a sum score as a predictor or an outcome variable within a structural model. Further, our proposed sum score model can be used to model the sum score as a variable that transmits all collective effects of or on the variables that make up the sum score. In this case, the sum score 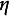 accounts for all covariances between its observed variables 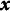 and other variables of the model 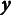 , i.e., 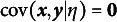 . In other words, the excrescent variables show no correlation with any other variable in the model. As a result, the covariance matrix of the sum score’s observed variables and all other variables of the model is of rank one, i.e., 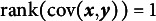 . We call this the *full transmission assumption*. As Grace and Bollen (2008) explained, for a composite with free weights, the effects of the components on the outcome variables, i.e., the collective effects, must be proportional, otherwise the effects of the composite on the outcome variables, i.e., the summary effects, will provide a distorted picture and the full transmission assumption will be violated.^4^ Consequently, the full transmission assumption depends on the scale of the variables forming the composite and the other variables in the model.

In the sum score context, the full transmission assumption implies that the collective effects of the components on an outcome variable must be equal. For our example model shown in Figure 2, the collective effects of the three variables 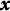 forming the sum score 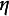 on the outcome variables 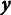 are calculated as follows: 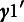 , where the two column vectors 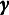 and 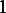 contain the two summary effects and ones, respectively. Note that when there is only one outcome variable, the components can always be scaled so that the full transmission assumption is satisfied. However, if there is more than one outcome variable in the model, this is no longer necessarily the case. Similar can be shown for the case in which a sum score is used to summarize the collective effects on the variables making up the sum score. The full transmission assumption offers researchers the opportunity to assess whether a sum score properly summarizes the collective effects. However, this assumption is in general not necessary for the use of sum scores in SEM, and its usefulness depends on the specific research context. Therefore, the following subsection shows how this assumption can be relaxed.

### 3.3 Relaxing the full transmission assumption in the sum score model

Our sum score model presented above implies that all covariances between the sum score’s observed variables, and other variables of the model are accounted for by the sum score. Since the weights are fixed in the sum score model, this assumption can be relaxed. For this purpose, the covariances between the excrescent variables and other exogenous variables of the model need to be specified as free model parameters. These covariances account for the covariation between the sum score’s observed variables and other variables of the model that the sum score does not account for. In other words, the covariances capture the covariances and/or effects that are not transmitted by the sum score. Relaxing the full transmission assumption allows us to mimic the results of the pseudo-indicator approach, as is also illustrated in Section 4. Therefore, this specification is particularly useful if the goal is to model sum scores without affecting the variance-covariance matrix implied by the target model (see also Rose et al., 2019). The target model is the model that does not contain the observed variables making up the sum scores, i.e., the model of the second step in the two-step approach.

**Figure 3:**
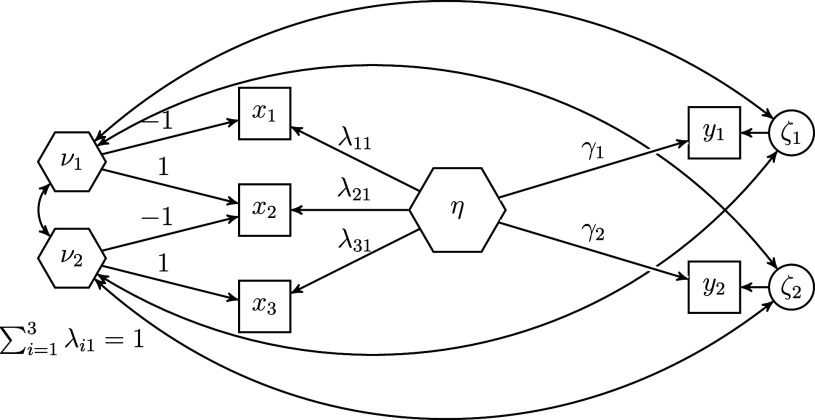
Example of a sum score model in which the full transmission assumption has been relaxed.

To demonstrate how the full transmission assumption can be relaxed, we consider the example model from Figure 2, where a researcher is studying the effect of a sum score on two observed outcome variables 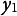 and 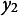 . As can be seen in Figure 3, to relax the assumption that the sum score fully transmits all collective effects of the observed variables making up the sum score on the two outcome variables 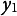 and 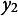 , we can specify free covariances between the excrescent variables 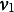 and 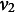 and the error terms 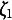 and 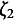 of the outcome variables. Note that the variances of the exogenous variables are omitted in the figure.

### 3.4 Taking into account random measurement error in the sum score model

In the sum score model, we assumed that the observed variables making up the sum score are free of random measurement error. However, this does not necessarily need to be the case, and in practice, the observed variables making up a sum score may be contaminated by random measurement error: 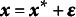 , where 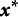 are the variables free from random measurement error, and 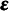 are the random measurement errors. The random measurement errors are assumed to be mutually uncorrelated and uncorrelated with 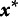 (and potential other variables of the model). In this case, the relationships between the sum score made up of measurement error contaminated variables and other variables of the model will most likely be distorted due to attenuation (Bollen & Lennox, 1991; Cohen et al., 1990). Similar is known from factor score regression in which composites are used as approximations for latent variables (e.g., Devlieger & Rosseel, 2017; Schuberth et al., 2023; Skrondal & Laake, 2001). To correct for random measurement error, we follow an approach frequently mentioned in the literature (Cole & Preacher, 2014; Hayduk, 1996; Hayduk & Littvay, 2012; Savalei, 2019), i.e., to model the random measurement error contaminated variable as a single indicator of a latent variable with a fixed error term variance. Consequently, for the sum score model random measurement error can be corrected in at least two ways: (1) on the sum score level or (2) on the observed variable level.^5^

To account for random measurement error on sum score level, the sum score can be modeled as a single indicator of a latent variable. Specifically, the loading of the sum score on the single-indicator latent variable is fixed to one, and the variance 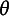 of the resulting error term 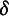 needs to be fixed to (1 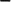 reliability of the sum score) 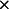 the variance of the sum score 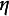 (Nunnally & Bernstein, 1994, Equation 7-6). In this way, the variance in the sum score that is due to random measurement error is partialled out and the variance of the latent variable accounts for the remaining variance in the sum score, i.e., the variance that is not caused by random measurement error.

**Figure 4:**
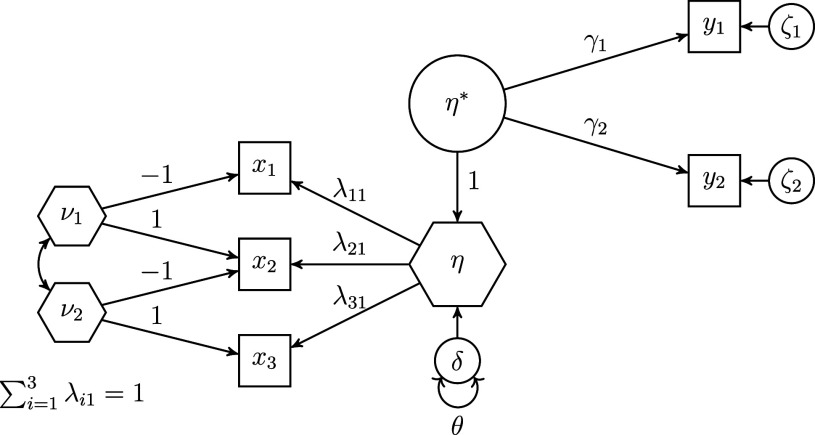
Accounting for random measurement error at the sum score level in the sum score model.

Figure 4 demonstrates this approach for our example model from Figure 2. If no correction takes place, i.e., if a researcher specifies the model from Figure 2, the collective effects of 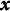 on 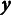 are calculated as: 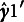 , where the estimated summary effects 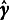 (and thus the collective effects) will likely be distorted due to attenuation. Since the sum score 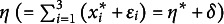 contains random measurement error, the estimated summary effects, i.e., the effect of 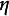 on 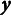 will converge in probability to 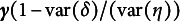 , where 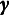 is the probability limit of the summary effects in case of no random measurement error. In contrast, if random measurement error is accounted for on sum score level, the estimated summary effects are calculated based on 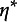 , i.e., the sum score corrected for random measurement error. Given a correct reliability estimate of the sum score, these estimated summary effects will converge in probability to 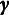 . Consequently, the collective effects are corrected for attenuation and equal the effects of 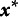 on 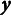 , i.e., the effects of the sum score’s components without random measurement error on the outcome variables.

Although correcting for measurement error on sum score level can address the issue of distorted path coefficient estimates between the sum score and the other variables of the model, the composite loadings and thus the free weights, i.e., the weights to form the excrescent variables, remain uncorrected because the correction takes place on sum score level. This is particularly problematic if a sum score is used to summarize the collective effects on its components.

To address this issue, we can take random measurement error on observed variable level into account. Specifically, each contaminated observed variable can be specified as a single indicator of a latent variable. The loading of the single indicator is fixed to one and the variance of the resulting error term is fixed to (1 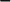 reliability of the observed variable) 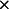 the variance of the observed variable. In this way, the latent variable captures the measurement error adjusted variance of the corresponding observed variable. As a result, the composites, i.e., the sum score and the excrescent variables, are formed from measurement error corrected variables, i.e., the single-indicator latent variables. Consequently, not only the relationships between the sum score and other variables of the model but also the relationships between the composites, i.e., the sum score and the excrescent variables, and their observed variables are corrected for attenuation. Hence, the composite loadings, and therefore the free weights, are corrected for random measurement error.

Taking random measurement error in the ways described above into account requires reliability estimates, which might be difficult to obtain in practice. If the variables making up a sum score are assumed to be unidimensional measures of a construct, Mosier’s (1943, Equation 5) formula can be used to determine the reliability of the sum score. Note that this way of correcting for random measurement error requires reliability estimates for each observed variable that makes up the sum score. Alternatively, Cronbach’s 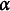 (Cronbach, 1951) can be used if the observed variables are assumed to be essential tau-equivalent measures of a construct. Moreover, although the proposed corrections can address potential attenuation bias in the parameter estimates given a correct reliability estimate, fixing the variance of an error term to a value derived from a data-based reliability estimate such as Cronbach’s 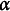 ignores the uncertainty in the reliability estimate. This carries the risk of drawing incorrect statistical inference (e.g., Oberski & Satorra, 2013). Furthermore, the two ways of correcting for random measurement error introduce additional variables. In the case of correcting for random measurement error on sum score level, the latent variable 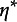 and the corresponding error term 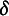 need to be specified (see Figure 4). However, as the effect of the latent variable on the sum score and the variance of the error term are fixed, no additional free parameters are added to the model. The same holds for the proposed correction on observed variable level. Consequently, the two approaches to accounting for random measurement error do not alter the number of free model parameters; thus, the model’s degrees of freedom remain unchanged. Yet, the fit of the model can be altered compared to the sum score model without a correction as the parameters are corrected for attenuation. Further, taking random measurement error into account does not limit the flexibility of our sum score model. Particularly, it is still possible to model a sum score as an outcome variable and to allow for covariances between the sum score and other variables of the model. Similarly, it is possible to relax the full transmission assumption, i.e., to allow for free covariances between the excrescent variables and other exogenous variables of the model.

## 4 Illustrative example

We demonstrate the capabilities of the sum score model based on the H–O specification by means of three scenarios. In each scenario, we make use of a different population model. Scenarios 1 and 2 present a situation in which a researcher uses a sum score to summarize the collective effects of or on the sum score’s components. In both scenarios, we use a population model with the same structure. However, in Scenario 2, the components making up the sum score are contaminated by random measurement error. This allows us to demonstrate how random measurement error can be taken into account on observed variable level in the sum score model. Finally, in Scenario 3, we apply the sum score model to a latent variable population model, i.e., sum scores are used as approximations for latent variables. This scenario allows us to demonstrate how random measurement error can be taken into account on sum score level in the sum score model.

In each scenario, we use the corresponding population variance-covariance matrix as input for the model estimation. Hence, strictly speaking, the model parameters were retrieved, not estimated. This allows us to provide explanations for the source of potential model misfit in the absence of sampling uncertainty. The exact model specifications used in each scenario are illustrated in Appendix A.2. All calculations were carried out in the statistical programming environment R (R Core Team, 2022). The different models were estimated using the full-information maximum likelihood estimator as implemented in the R package lavaan (Rosseel, 2012) based on 200 observations.^6^

### 4.1 Scenario 1: Comparison of approaches to deal with sum scores

**Figure 5:**
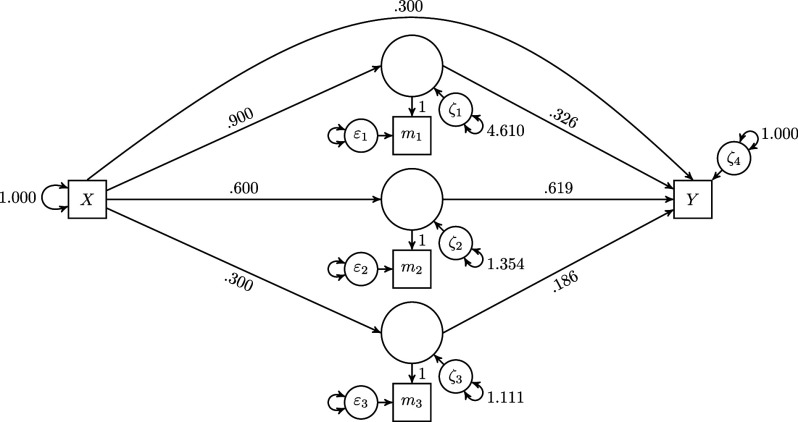
Population model used in Scenarios 1 and 2.

In the first scenario, we consider the population model shown in Figure 5.

This population model consists of 5 observed variables, i.e., one exogenous variable *X*, three mediator variables 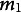 to 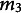 , and one outcome variable *Y*. All observed variables are mean centered. The collective effects of *X* on the three mediator variables are 0.9, 0.6, and 0.3, and the collective effects of the mediator variables on the outcome variable *Y* are 0.326, 0.619, and 0.186. The variance of *X* is set to 1, and the variances of error terms 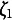 to 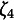 are set to 4.610, 1.354, 1.111, and 1.000, respectively. Although not shown in Figure 5, the error terms of the mediator variables are allowed to covary as follows: 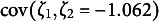 , 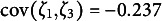 , and cov( 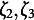 )=0.222. Finally, in this scenario, we assume that all variables are free from random measurement error. Therefore, the variances of the random measurement errors 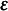 are set to 0, i.e., var( 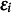 )=0 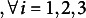 .

To summarize the collective effects on and of the three mediator variables 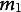 , 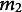 , and 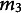 , a researcher replaces them by a sum score. This could, for example, represent a situation in which a researcher studies a mother’s availability to interact with and monitor her children. Specifically, the mother’s availability can be regarded as the sum of the number of children, the mother’s illness, and hours of maternal employment (Cohen et al., 1990). For this purpose, the researcher used the following four approaches:

1. The sum score model assuming full transmission,
2. The sum score model not assuming full transmission,
3. The pseudo-indicator approach using unit weights, and
4. The two-step approach, which is the conventional way of dealing with sum scores in SEM, i.e., in the first step the sum score is created, and second, the sum score is used in a path analysis together with the other variables.

In addition, as a fifth approach, we consider the refined H–O specification. In this specification, the mediator variables 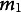 to 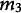 form a composite of which the weights are freely estimated. We did not include the one-step approach, i.e., we did not use the approach in which the sum score is modeled as a formatively measured latent variable because this approach does not permit modeling a sum score as an outcome variable.

Table 1 shows the results of the five approaches. For all approaches using the sum score, i.e., Approaches 1) to 4) above, the weights were equal to one. In contrast, in the refined H–O specification, where 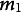 , 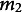 , and 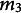 , instead of a sum score, form a composite with free weights, we obtained the following weights: 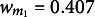 , 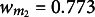 , and 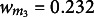 . Also, Table 1 reports the results for the two summary effects, i.e., the path coefficient estimates between *X* and *M*, and *M* and *Y*, the direct effect of *X* on *Y*, the collective effects of *X* on 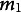 to 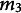 and 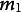 to 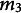 on *Y*, and various model fit statistics, i.e., 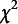 -test statistic with its degrees of freedom (df), the root mean square error of approximation (RMSEA, Hu & Bentler, 1999), and the standardized root mean square residual (SRMR, Hu & Bentler, 1998). The collective effects are no model parameters in the different sum score models and the refined H–O specifications, but they can be derived as indirect effects of *X* on 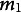 to 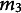 , and 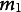 to 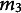 on *Y*, respectively. The values in parentheses show the population values of the parameters (see also Figure 5). For the two-step approach, the collective effects cannot be derived as the mediator variables are not part of the model of the second step. Similarly, the collective effects are not reported for the pseudo-indicator approach.

**Table 1:** Results of Scenario 1

Specification:	(1) Sum score		(2) Sum score		(3)		(4) Two-step		(5) Refined H–O	
	model assuming		model not assuming		Pseudo-indicator		approach		specification	
	full transmission		full transmission		approach					
	Est.	SE	Est.	SE	Est.	SE	Est.	SE	Est.	SE
*Weights*										
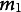	1.000	NA	1.000	NA	1.000	NA	1.000	NA	0.407	0.065
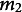	1.000	NA	1.000	NA	1.000	NA	1.000	NA	0.773	0.086
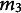	1.000	NA	1.000	NA	1.000	NA	1.000	NA	0.232	0.068
*Summary effects*										
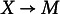	1.800	0.157	1.800	0.157	1.800	0.157	1.800	0.157	0.900	0.131
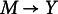	0.325	0.034	0.325	0.034	0.325	0.034	0.325	0.034	0.800	0.121
*Direct effect*										
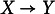 (0.300)	0.435	0.096	0.435	0.096	0.435	0.096	0.435	0.096	0.300	0.095
*Collective effects*										
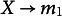 (0.900)	1.800	0.157	1.800	0.157	NA	NA	NA	NA	0.900	0.131
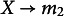 (0.600)	0.352	0.061	0.188	0.067	NA	NA	NA	NA	0.600	0.073
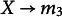 (0.300)	0.361	0.052	0.401	0.064	NA	NA	NA	NA	0.300	0.063
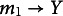 (0.326)	0.325	0.034	0.325	0.034	NA	NA	NA	NA	0.326	0.033
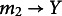 (0.619)	0.325	0.034	0.325	0.034	NA	NA	NA	NA	0.619	0.063
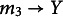 (0.186)	0.325	0.034	0.325	0.034	NA	NA	NA	NA	0.186	0.053
*Model Fit*										
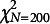	40.154		0.000		0.000		0.000		0.000	
df	4		0		0		0		2	
RMSEA	0.213		0.000		0.000		0.000		0.000	
SRMR	0.085		0.000		0.000		0.000		0.000	

*Note*: The refined H–O specification does not create sum scores, but weighted composites; it is listed for comparative reasons only.

As can be seen from Table 1, all approaches using sum scores, i.e., Approaches (1) to (4), produced the same direct effect of *X* on *Y* and the same summary effects of *X* on *M*, and *M* on *Y*, i.e., 0.435, 1.800, and 0.325, respectively. Similarly, the corresponding standard errors (SEs) were the same. However, the approaches differed in respect of the model fit statistics, i.e., 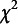 -test statistic, RMSEA, SRMR, and the collective effects.

The sum score model that does not assume full transmission showed no misfit, and in fact, it showed the exact same 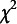 -test statistic, number of df, and the RMSEA value as the pseudo-indicator approach and the two-step approach. This was expected as this sum score model emulates the pseudo-indicator approach which was designed to model the sum score in such a way that its inclusion does not affect the model-implied variance-covariance matrix of the target model (Rose et al., 2019).^7^ In our example, the target model is identical to the model of the second step of the two-step approach. Since this model is saturated, all three approaches show a perfect fit.

Considering the sum score model that assumes full transmission, the various model fit criteria showed a misfit. This is because the sum score does not fully transmit the collective effects of and on its components. This is also evidenced by the derived collective effects, which differ from the collective effects in the population model. Consequently, a researcher would likely draw the wrong conclusions from the summary effects. Finally, the refined H–O specification could perfectly reproduce the variables’ variance-covariance matrix as the overall model fit criteria highlight. Moreover, the derived collective effects are identical to the ones of the population model. Consequently, although it was not possible to properly summarize the collective effects of the mediator variables using a sum score, allowing for different weights, the collective effects could be properly summarized.

### 4.2 Scenario 2: Correcting for random measurement error on the observed variable level

Scenario 2 demonstrates how random measurement error can be taken into account on the observed variable level in the sum score model. For this reason, we use the population model of Scenario 1, see Figure 5. However, and in contrast to Scenario 1, in this scenario, the mediator variables 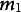 , 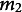 , and 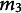 are contaminated by random measurement error, i.e., the variances of the random measurement errors 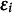 are positive. Specifically, we contaminated each mediator variable by random measurement error in such a way that the reliabilities of the mediator variables are 0.935, 0.852, and 0.659, respectively. In this scenario, we consider the following three approaches:

1. The sum score model assuming full transmission and not taking measurement error into account,
2. The sum score model assuming full transmission and taking random measurement error into account on the observed variable level, and
3. The refined H–O specification with free weights, thus assuming full transmission, and taking random measurement error on the observed variable level into account.

The Approaches (7) and (8) take random measurement on the observed variable level into account. For this purpose, we model each mediator variable as a single indicator of a latent variable, as described in Subsection 3.4. Specifically, we use the population reliabilities and therefore fix the variances of the resulting error terms to 0.38, 0.3, and 0.6276. As a result, the sum score and composite, respectively, are made up of random measurement error-corrected variables. In contrast, in the sum score model that assumes full transmission and does not take random measurement into account, i.e., in Approach 6), the sum score is made up of the original mediator variables. Consequently, due to attenuation, its parameter estimates are expected to be biased.

Table 2 shows the results for the three approaches. The two sum score models yielded unit weights and the refined H–O specification produced weights of 0.407, 0.773, and 0.232 for 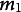 , 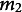 , and 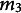 , respectively. The sum score model that does not account for random measurement error, i.e., Approach (6), produced biased parameter estimates, i.e., a biased direct effect of *X* on *Y* and biased collective effects. In addition, the model fit criteria showed a model misspecification, which is caused by attenuation and the fact that the sum score cannot fully transmit the collective effects. Similarly, although Approach (7) corrects for random measurement error, it produced biased parameter estimates. As in Scenario 1, this is because the sum score cannot fully transmit the collective effects. This is also evidenced by the various model fit criteria which indicate a model misfit. Finally, the refined H–O specification taking random measurement error into account was able to retrieve the population parameters, and the model fit criteria showed no misfit.

**Table 2:** Results of Scenario 2

Specification:	(6) Sum score model		(7) Sum score model		(8) Refined H–O	
	assuming full		assuming full		specification	
	transmission & not		transmission &		& correcting for random	
	correcting for random		correcting for random		measurement error on	
	measurement error		measurement error on the		the observed	
			observed variable level		variable level	
	Est.	SE	Est.	SE	Est.	SE
*Weights*						
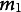	1.000	NA	1.000	NA	0.407	0.069
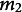	1.000	NA	1.000	NA	0.773	0.096
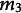	1.000	NA	1.000	NA	0.232	0.094
*Summary effects*						
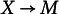	1.800	0.176	1.813	0.175	0.900	0.137
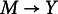	0.257	0.031	0.333	0.040	0.800	0.136
*Direct effect*						
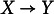 (0.300)	0.558	0.097	0.417	0.107	0.300	0.110
*Collective effects*						
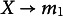 (0.900)	1.800	0.176	1.813	0.175	0.900	0.137
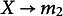 (0.600)	0.360	0.064	0.402	0.073	0.600	0.081
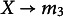 (0.300)	0.430	0.063	0.351	0.066	0.300	0.078
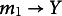 (0.326)	0.257	0.031	0.333	0.040	0.326	0.040
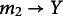 (0.619)	0.257	0.031	0.333	0.040	0.619	0.080
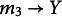 (0.186)	0.257	0.031	0.333	0.040	0.186	0.077
*Model Fit*						
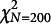	30.426		25.199		0.000	
df	4		4		2	
SRMR	0.079		0.066		0.000	
RMSEA	0.182		0.163		0.000	

*Note*: The refined H–O specification does not create sum scores, but weighted composites; it is listed for comparative reasons only.

### 4.3 Scenario 3: Correcting for random measurement error on the sum score level

**Figure 6:**
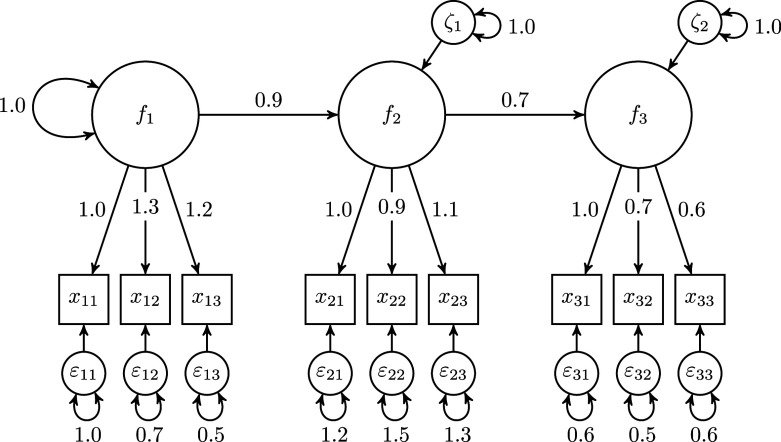
Population model used in Scenario 3.

In the third scenario, we consider a latent variable population model as depicted in Figure 6. This population model consists of three latent variables 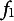 , 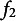 , and 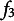 , where each is measured by three observed variables. The values of the population parameters are given in the figure.

In this scenario, the researcher uses sum scores to approximate the latent variables. Therefore, the researcher’s main interest is in studying the relationships between the latent variables and not in summarizing collective effects. For this reason, we relax the full transmission assumption in all sum score models. In particular, we consider the following four approaches:

1. The sum score model not assuming full transmission,
2. The two-step approach,
3. The sum score model not assuming full transmission and taking random measurement error on the sum score level into account, and
4. The two-step approach with a correction for random measurement error.

The Approaches (9) and (10) and the Approaches (11) and (12), respectively, are expected to produce the same results. For the sum score model and the two-step approach that do not correct for random measurement error, the estimated relationships between the latent variables are expected to be biased due to attenuation (Cohen et al., 1990; Schuberth et al., 2023). In contrast, Approaches (11) and (12) correct for random measurement error on the sum score level, i.e., each sum score is modeled as a single indicator of a latent variable with a fixed loading and error term’s variance. For more details, see Section 3.4 above. We have not included the pseudo-indicator approach in this scenario as it produces the exact same results as the sum score model that does not assume full transmission. Similarly, we have not included the one-step approach as it does not permit modeling a sum score as an outcome variable.

Table 3 presents the results for the various approaches. The sum score model and the two-step approach that do not correct for random measurement error produced the same standardized path coefficient estimates. As expected, the estimated standardized path coefficient estimates differed from the standardized population path coefficients because of attenuation. Also, the 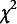 -test statistic, the df, and the RMSEA were the same for the two approaches, indicating a model misfit. The SRMR differed for the two approaches because the number of residuals is different. Similarly, the sum score model and the two-step approach that correct for random measurement error showed the same results. However, in this case, the distortion in the standardized path coefficient estimates diminished when random measurement error was taken into account. Finally, the model fit criteria indicated no misfit.

**Table 3:** Results of Scenario 3

Specification:	(9) Sum score		(10) Two-step		(11) Sum score		(12) Two-step	
	model not assuming		approach not		model relaxing full		approach	
	full transmission &		correcting for		transmission &		correcting for	
	not correcting		random		correcting for random		random	
	for random		measurement		measurement error		measurement	
	measurement error		error		on the sum score level		error	
*Standardized direct effects*	Est.	SE	Est.	SE	Est.	SE	Est.	SE
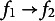 (0.669)	0.552	0.049	0.552	0.045	0.669	0.054	0.669	0.054
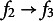 (0.686)	0.568	0.048	0.568	0.048	0.686	0.052	0.686	0.052
*Model Fit*								
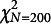	2.530		2.530		0.000		0.000	
df	1		1		1		1	
RMSEA	0.087		0.087		0.000		0.000	
SRMR	0.026		0.031		0.000		0.000	

## 5 Discussion

Traditionally, sum scores are studied in SEM following a two-step procedure, which omits the creation of the sum score from the model and, therefore, does not permit researchers to rigorously assess their sum scores and exploit SEM’s full potential. More recent approaches to model sum scores in structural equation models address some of the drawbacks of the traditional approach (Grace & Bollen, 2008; Rose et al., 2019). However, they also show some limitations. Particularly, the one-step approach shows limited flexibility in modeling sum scores in a structural equation model. Moreover, the literature on the pseudo-indicator approach currently lacks guidance on modeling a sum score that fully transmits the collective effects on its components, making it difficult for applied researchers to generally assess the full transmission assumption using this approach.

To address this issue, we introduce the sum score model based on the refined H–O specification. The sum score model overcomes the limitations of the existing approaches. First, our sum score model explicates the creation of a sum score, i.e., it models the sum score, and allows us to specify a sum score as an outcome variable in the structural model. Thus, it overcomes the limitations of both the two-step approach and the one-step approach, which either do not model sum scores or have limited flexibility in modeling sum scores. Second, as our illustrative example shows, our sum score model can mimic the results of the pseudo-indicator approach, which was proposed to emulate the results of the two-step approach, and thus offers all the advantages of the pseudo-indicator approach. Particularly, it allows researchers to include a sum score retaining its components in the model without affecting the model-implied variance-covariance matrix of the target model, i.e., the model that contains the sum score and other variables of interest but not the sum score’s components. Third, our sum score model offers researchers the opportunity to assess whether the sum score fully transmits the collective effects of or on the variables that make up a sum score. This is not possible with the two-step approach because the components of a sum score are not modeled in this approach. Similarly, the one-step approach is limited in this regard because it does not allow a sum score to be modeled as a dependent variable. Moreover, considering the pseudo-indicator approach, Rose et al. (2019, p. 6) mention that “some of the rules may be relaxed to simplify the model or to consider specific assumptions.” In addition, they provide an example in which they fix the covariances between the components of a sum score and the measurement error variances of the indicators of one or more latent variables to zero to ensure that the covariances between the sum scores’ components and the latent variables’ indicators are fully accounted for by the sum score. Although this way of specifying the covariance allows for modeling a sum score that fully transmits the collective effects of its components, there is currently no guidance on how to constrain these covariances to ensure that a sum score fully transmits the collective effects on its components. It is up to future research to show whether it is possible to assess the full transmission assumption in general using the pseudo-indicator approach and, if so, how it should be done. Note that the usefulness of assessing the full transmission assumption depends on the specific research context and is not a requirement for using composite scores including sum scores in SEM. Therefore, researchers are encouraged to carefully consider the exact specification of the sum score model. Fourth, the H–O specification allows us to freely estimate weights, which gives researchers more flexibility and overcomes a further limitation of the pseudo-indicator approach. Although our illustrative example and first studies provide arguments for using composites with free weights (e.g., Grace & Bollen, 2008; Heise, 1972), future research needs to provide sophisticated guidelines and further recommendations as to when free weights should be preferred over fixed weights such as unit weights. Against this background, the sum score model based on the H–O specification allows researchers to better judge and defend the use of sum scores. They can do this empirically, by means e.g., of model comparisons using a chi square difference test or information criteria such as the Akaike information criterion (AIC, Akaike, 1998) or the Bayesian information criterion (BIC, Schwarz, 1978), as well as conceptually, in that researchers can better understand whether the model containing sum scores reflects their theoretical arguments. Table 4 juxtaposes the properties of the different approaches for dealing with sum scores in SEM.

**Table 4:** Properties of the different approaches for dealing with sum scores in SEM

	Two-step	One-step	Pseudo-indicator	Sum score model
	approach	approach	approach	based on H–O
				specification
Allows for...				
...taking the sum score’s formation into account	✗	✓	✓	✓
...modeling a sum score as an outcome variable	✓	✗	✓	✓
...assessing whether the sum score fully transmits the effects of or on its components	✗	🔾	?	✓
...freely estimating the weights of a composite	✗	✓	✗	✓

*Note*: ✓: possible; 🔾: limitedly possible; ✗: not possible; ?: currently unclear

Further, our study presents two ways of accounting for random measurement error in the sum score model, i.e., on the observed variable or the sum score level. Note that these ways of dealing with random measurement errors are not unique to our sum score model, but can also be applied to the other approaches presented. Accounting for random measurement error allows researchers to address attenuation bias in the parameter estimates (Cohen et al., 1990). For this purpose, reliability estimates of the variables that make up a sum score or a reliability estimate of the sum score are required to perform these corrections. Empirical researchers can obtain such reliability estimates from existing literature, or they could use the test-retest method (Guttman, 1945) to estimate the reliability of an observed variable or a sum score. Further, a closed-form formula for McDonald’s (1999) 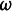 can be used to estimate the reliability of a sum score under unidimensionality without running a confirmatory factor analysis (Hancock & An, 2020). Similarly, Cronbach’s 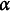 can be used to determine the reliability of a sum score if its variables are essential tau-equivalent measures (Novick & Lewis, 1967).^8^ Alternatively, if various measures for the variables that make up a sum score are available, the original variables can be replaced by latent variables using the measures as indicators. If possible, this approach is preferred because the researcher does not risk incorrect statistical inference by ignoring uncertainty in the reliability estimate (Oberski & Satorra, 2013).

A potential drawback of the presented sum score model could be its complexity as it additionally introduces new composites, i.e., the excrescent variables. Compared to the two-step approach, which omits the creation of the sum score from the model, this is certainly true. However, the two-step approach sacrifices technical rigor in favor of practicality (Li & Calantone, 1998). In comparison to the one-step approach, our sum score model is not more complex. Although additional variables need to be specified in the sum score model, the number of free model parameters and thus degrees of freedom remain the same. While in the one-step approach, the variances and covariances of the variables that make up a sum score are free model parameters, in our sum score model, these parameters are replaced by the same number of free model parameters, i.e., variances of the excrescent variables and the sum score, covariances between the excrescent variables, and composite loadings. Note that the use of more complicated parameterization to achieve a specific goal is not uncommon in SEM. For instance, Rindskopf (1984) introduced phantom and imaginary variables to model equality and inequality constraints in SEM.

A limitation of our sum score model is that we followed the SEM framework proposed by Jöreskog (1970), and we, therefore, assumed that the observed variables of a model, including those making up a sum score, follow a multivariate normal distribution. However, in empirical studies, this assumption is likely to be violated. To account for this fact, ML estimation with robust standard errors and test statistics could be used (e.g., Satorra & Bentler, 1994). Further, the observed variables are often categorical in empirical research, e.g., as responses to five or seven-point Likert scales (Rhemtulla et al., 2012). In such cases, treating the observed variables as continuous could lead to biased parameter estimates (Johnson & Creech, 1983). Although weighted least squares estimators have been proposed in the SEM context to deal with categorical observed variables (e.g., Lee et al., 1990; Muthén, 1984), future research needs to show whether these estimators are compatible with the sum score model.

## Figures and Tables

**Figure A1:**
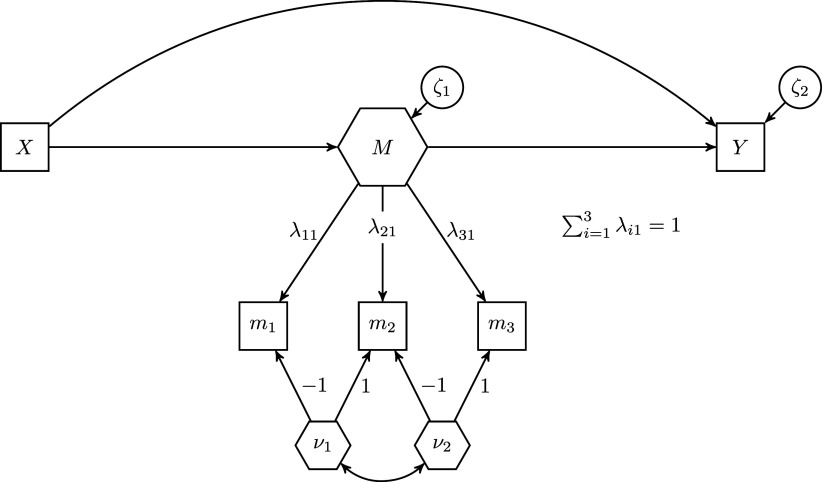
Approach 1) The sum score model assuming full transmission.

**Figure A2:**
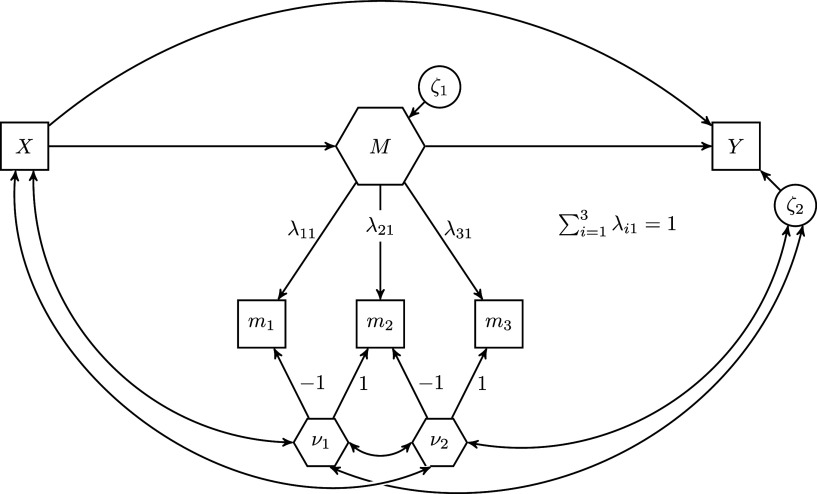
Approach 2) The sum score model relaxing the full transmission assumption.

**Figure A3:**
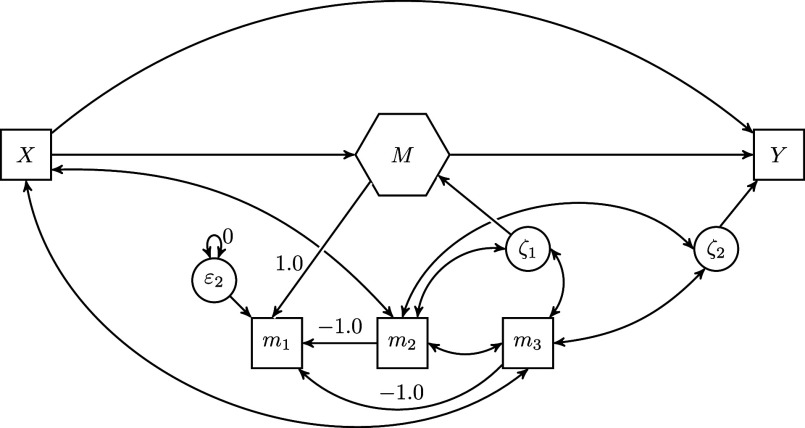
Approach 3) The pseudo-indicator approach using unit weights.

**Figure A4:**
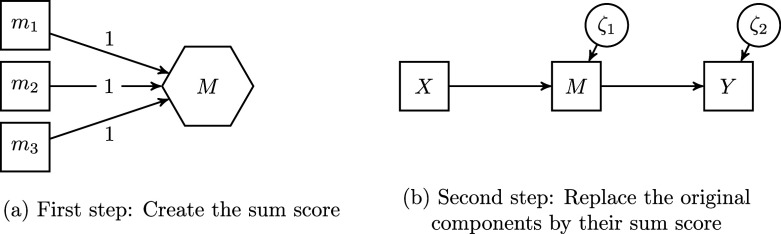
Approach 4) The two-step approach.

**Figure A5:**
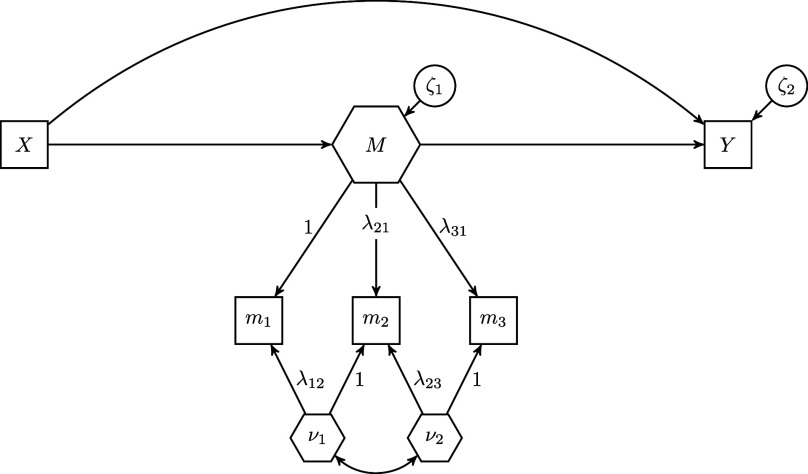
Approach 5) The refined H–O specification.

**Figure A6:**
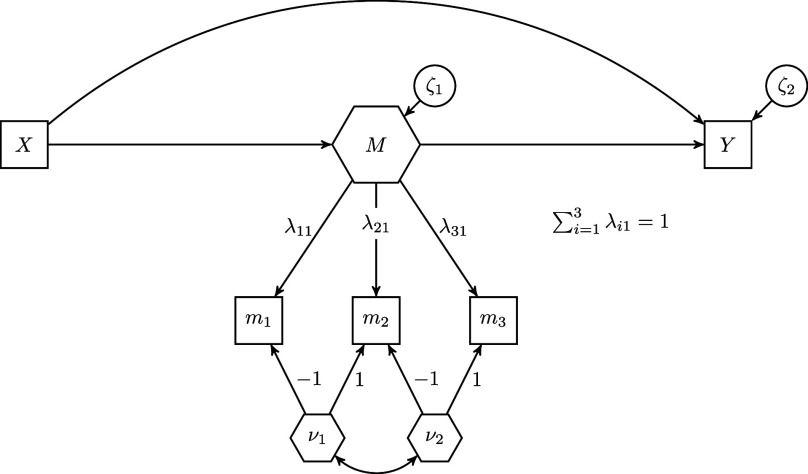
Approach 6) The sum score model assuming full transmission and not taking measurement error into account.

**Figure A7:**
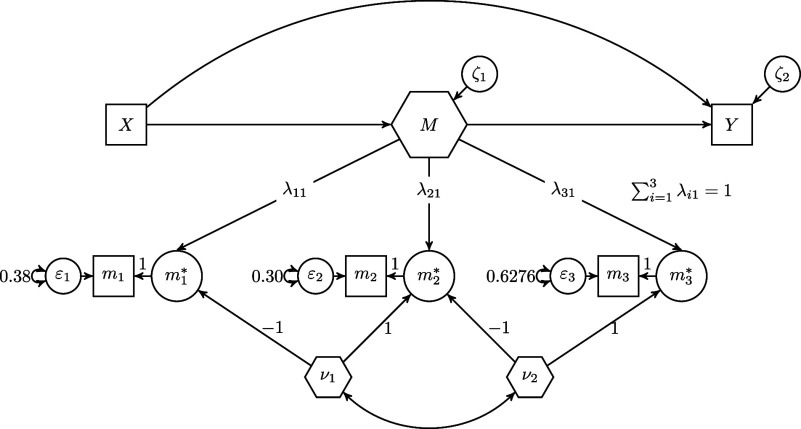
Approach 7) The sum score model assuming full transmission and taking random measurement error into account on the observed variable level.

**Figure A8:**
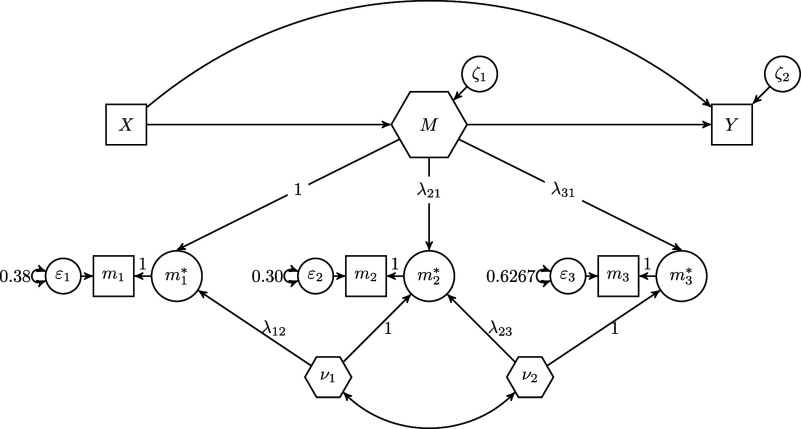
Approach 8) The refined H–O specification taking into account random measurement error on the observed variable level.

**Figure A9:**
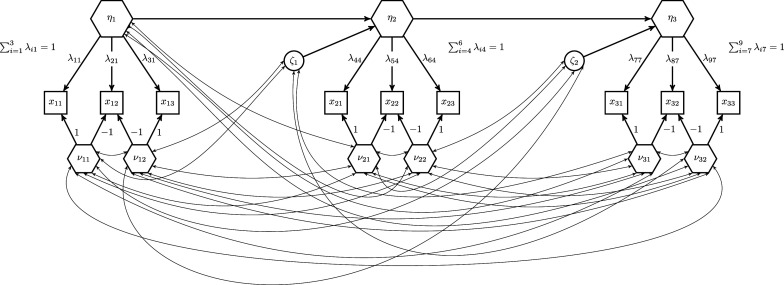
Approach 9) The sum score model relaxing the full transmission assumption.

**Figure A10:**
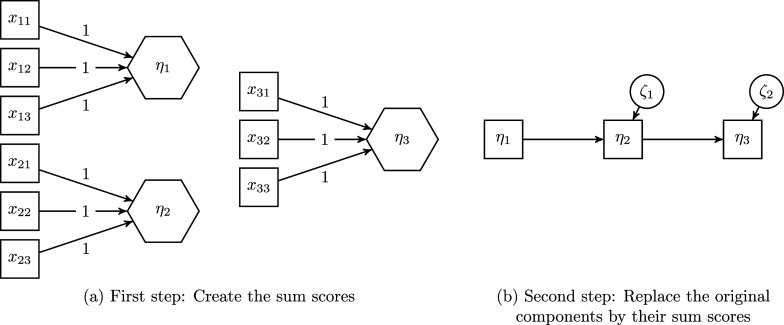
Approach 10) The two-step approach.

**Figure A11:**
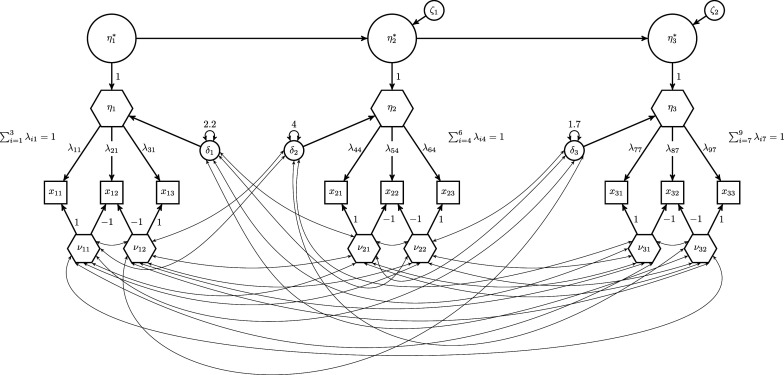
Approach 11) The sum score model relaxing the full transmission assumption and taking into account random measurement error on the sum score level.

**Figure A12:**
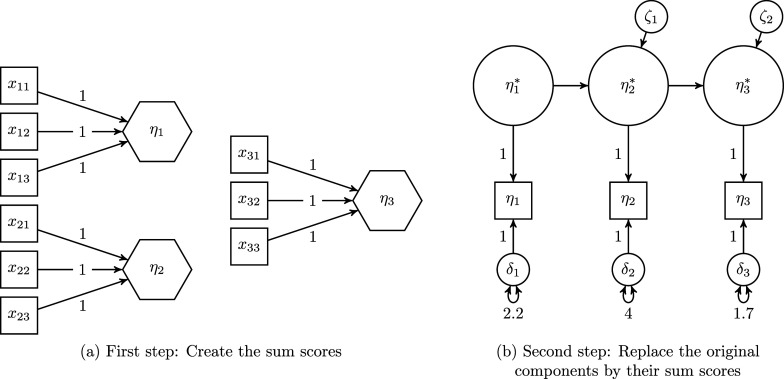
Approach 12) The two-step approach with a correction for random measurement error.

## Data Availability

The results of the illustrative example including the R code to reproduce the results are available in the Open Science Framework repository at https://osf.io/y3m4r/?view_only=e2b016994d764bd28645c346a933409d

## Footnotes

1 More recently, it has been proposed to calculate a sum score as the factor score for a parallel measurement model (McNeish & Wolf, 2020). This approach could also be considered a two-step approach, as the first step is to extract the factor scores, and the second step is to study the relationships between the factor scores and potential other variables. Although the factor score extracted from a parallel measurement model is perfectly correlated with the sum score, the factor score is not necessarily identical to the sum score because the factor score is likely to have a different variance depending on the parameterization of the parallel measurement model. This was also recognized by McNeish and Wolf (2020), who recommend the pseudo-indicator approach (Rose et al., 2019) for researchers interested in obtaining scores that are exactly equal to sum scores. As the pseudo-indicator approach is discussed in our paper, we do not discuss the approach based on the parallel measurement model.

2 We assume that the observed variables are not perfectly linearly dependent, i.e., their variance-covariance matrix  is of full rank.

3 As can be seen immediately, if the sum of the loadings of the composite of interest was fixed to the number of variables that make up the composite, the composite of interest would be the average of its components.

4 In the case of free weights, this assumption cannot be violated if there is only one other variable in the model besides the composite and its components.

5 In the literature on causal-formative measurement models, it has been proposed to partition the causal indicators into at least two composites and to use these composites as reflective indicators of a latent variable to allow for random measurement error in the composites (Treiblmaier et al., 2011). Although this approach no longer models the sum score as a single variable, this approach is also conceivable for our sum score model. For more details, we refer the interested reader to the original study of Treiblmaier et al. (2011).

6 The complete R code and the results can be accessed via the following link: https://osf.io/y3m4r/?view_only=e2b016994d764bd28645c346a933409d.

7 As Rose et al. (2019) explained, the pseudo-indicator approach, and thus the sum score model with relaxed full transmission assumption, produces the exact same  test statistic, df and RMSEA as the target model. However, between the target model and the pseudo-indicator approach the SRMR value can differ because the number of residuals is different. To ensure that the pseudo-indicator approach and the sum score model produce the SRMR of the target model, the calculation of the SRMR needs to be adjusted (Rose et al., 2019).

8 Otherwise, if these variables are congeneric measures, Cronbach’s  still provides a lower bound estimate for the reliability of a sum score (Sijtsma, 2009).

## APPENDIX

### A.1 Collective effects for the example model from Figure 1

In the following, we derive the collective effects of the observed variables  making up the composite of interest  on the outcome variables  for the example model shown in Figure 1. For this example model, the collective effects are calculated as the coefficients of the regression  on  , i.e.,  , where  ,  and  . For the sake of simplicity, it is assumed that all variables are mean centered. The regression coefficients are calculated as follows:

(A.1)

(A.2)

(A.3)

(A.4)

(A.5)

(A.6)

(A.7)

(A.8)
